# Exploring the Y-Balance-Test scores and inter-limb asymmetry in soccer players: differences between competitive level and field positions

**DOI:** 10.1186/s13102-022-00438-w

**Published:** 2022-03-23

**Authors:** Francisco Tomás González-Fernández, Luis Manuel Martínez-Aranda, Moisés Falces-Prieto, Hadi Nobari, Filipe Manuel Clemente

**Affiliations:** 1 Department of Physical Education and Sport, Faculty of Education and Sport Sciences, Campus of Melilla, University of Granada, 52006 Melilla, Spain; 2 SER Research Group, CESAG, Pontifical University of Comillas, 07013 Palma, Spain; 3 Faculty of Sport, Catholic University of Murcia (UCAM), 30107 Murcia, Spain; 4 Human Movement Neuroscience Research Group (Neuromove), UCAM, 30107 Murcia, Spain; 5 Research Center High Performance Soccer, Marcet Academy, 08035 Barcelona, Spain; 6 Department of Exercise Physiology, Faculty of Educational Sciences and Psychology, University of Mohaghegh Ardabili, 56199-11367 Ardabil, Iran; 7 Department of Physiology, Faculty of Sport Sciences, University of Extremadura, 10003 Cáceres, Spain; 8 Sports Scientist, Sepahan Football Club, 81887‐78473 Isfahan, Iran; 9 Escola Superior Desporto e Lazer, Instituto Politécnico de Viana do Castelo, Rua Escola Industrial e Comercial de Nun’Álvares, 4900-347 Viana do Castelo, Portugal; 10 Instituto de Telecomunicações, Delegação da Covilhã, 1049-001 Lisboa, Portugal

**Keywords:** Y-balance, Assessment, Range of motion, Postural control, Soccer players

## Abstract

**Background:**

The postural stability seems to be important in the physical development of the soccer player and the specific tasks related to the game. In addition, it is related to the injury risk and therefore, with the injury prevention and retraining processes. In this context, the Y Balance Test (YBT) is presented as a tool to assess dynamic postural control.

**Objective:**

This study aimed to explore the differences and possible correlations in the YBT scores and inter-limb asymmetry for anterior (ANT), posteromedial (PM) and posterolateral (PL) directions by category and field position in soccer players.

**Methods:**

173 males soccer players aged between 14 and 33 years old agreed to participate. Five categories and six field position were considered in this study. A standardised protocol was used at multiple clubs during the pre-season assessment of musculoskeletal function in soccer players. All the players performed the Y Balance Test (YBT) (official YBT Kit), assessing the dominant and non-dominant leg for three YBT directions (anterior-AN, posteromedial-PM and posterolateral-PL), inter-limb asymmetry and composite score.

**Results:**

For AN, amateur and semiprofessional obtained the highest values for Dominant and Non-Dominant legs (Range_mean_ = 101.8–109.4%) and the lowest level in PRO players (mean: 62.0%). Concerning PM-PL, semiprofessional (Range_mean_ = 126.4–132.7%, dominant and non-Dominant respectively), followed by professional and amateur reported higher scores compared to youth categories. Inter-limb asymmetry showed higher values in lower age categories. The best composite scores were detected in semiprofessionals (Range_mean_ = 113.3–126.7% for dominant and Range_mean_ = 113.8–129.7% for non-Dominant leg), compared with the rest of the categories and for each field position evaluated.

**Conclusion:**

Comparisons between field-positions revealed that centre-backs were worse than wingers and forwards. In order to explain variations in dynamic balance between competitive levels within the same age-group, special considerations about training programmes and related co-variables should be considered.

**Supplementary Information:**

The online version contains supplementary material available at 10.1186/s13102-022-00438-w.

## Introduction

Over the last 10 years, the assessment of postural control has become increasingly attractive to the sports scientist for the injury and recurrence prevention (mainly in the lower limbs) [1–4], in different sporting activities. In this sense, there is also abundant evidence related to biomechanical principles of postural stability [5]. However, most balance research has been conducted in clinical settings while there are scarce reports related to sport-specific balance [5]. Accordingly, postural balance is required to maintain the stability during game development [6]. Nevertheless, balance training is often neglected, despite its role in reducing injuries and their recurrence [7]. In fact, during soccer practice and games, passing and kicking are the most frequently used playing techniques, preferably performed with the dominant leg while the non-dominant leg is used as standing leg [7].

As a consequence, postural control in the standing leg might be superior compared to the kicking leg. This leg difference in unipedal balance performance may be further enhanced based on athletes’ soccer experience (i.e., years of soccer training) [8]. For this reason, postural control requires mastery of the body in space for stability and orientation purposes [6, 9]. Postural stability is defined as the ability to maintain the centre of body mass within the support base [9] and explicitly refers to the ability to maintain a correct relationship between one's body segments and the environment in order to perform a task [7]. This postural control is maintained due to the dynamic integration of internal and external forces regulated by visual, vestibular and somatosensory stimuli, i.e. by implementing different neuromuscular control strategies [9–11].

Numerous research studies have highlighted the importance of postural stability in the physical development of the soccer player and its relationship with specific tasks related to the game [12–14], as well as its relationship with injury prevention and retraining. For this reason, before the start of the season it is necessary to evaluate in order to optimise the performance of the different player-specific positions, as well as to determine the return to play after injury [14–16].

The Y Balance Test (YBT) is a tool to assess dynamic postural control. In fact, this tool is a variation of the Star Excursion Balance Test (SEBT) that minimises the initial test from eight directions to three: anterior (AN), posteromedial (PM) and posterolateral (PL) [14, 17–19]. The YBT shows its efficacy as a valid and reliable tool for predicting future lower limb injuries [14, 20–22]. Moreover, the test does not involve an additional cost, since it can be performed with the usual training equipment, although there is specific equipment for its assessment, such as the Y Balance Test®. This functional test requires strength, flexibility, neuromuscular control, stability, range of motion, balance and proprioception [14]. The YBT is an interesting tool, but it should be considered that the YBT should not be a cause-effect justification for the appearance of injuries, since these have a multifactorial origin [8, 13].

Traditionally, postural control training is neglected, even though the literature has shown that its application as a strategy reduces injuries and recurrence [5, 8]. This work should therefore be rapidly integrated into comprehensive programmes (eccentric strength, pelvic core stability, range of motion, etc.) in order to improve effectiveness with the aim of reducing injuries. Indeed, in official competitions, when athletes perform different extreme sport skills, the competence to maintain a stable position is a key factor not only for a successful skill application, but to also reduce the likelihood of injuries [23]. Therefore, it may be of great interest to test and control the dynamic stability of soccer players. In addition, YBT has been associated with performance and injury prevention in soccer players, where few studies have investigated the differences in dynamic balance abilities among different aged soccer players [14]. Currently, the relationship between semi-professional and professional level of soccer player and YBT scores is unknown. Consequently, given this gap in the literature, this study aimed to explore the differences and possible correlations in the YBT scores and inter-limb asymmetry for AN, PM and PL by category and field position in soccer players. In line with previous studies, we expected that players at higher categories would show better scores in this specific test as well as field positions related to striking (wingers of forwards) compared to lower field lines.

## Material and methods

### Participants

173 male soccer players with more than 5 years (yrs) of regular sport practice participated in this study. Five different categories were considered: Under 16 (U16) [n = 48; age = 14.18 ± 2.02 yrs, height = 171.09 ± 2.02 cm]; Under 19 (U19) [n = 62; age = 18.20 ± 2.04 yrs, height = 172.37 ± 8.26 cm]; Amateur [n = 21; age = 25.42 ± 4.52 yrs, height = 180.09 ± 5.29 cm]; Semi-professional (SPRO) [n = 19; age = 25.68 ± 3.00 yrs, height = 180.00 ± 5.93 cm]; Professional (PRO) [n = 23, age = 32.91 ± 3.04 yrs, height = 180.13 ± 5.35 cm)]. All soccer players competed on a federated basis under the jurisdiction of the Royal Spanish Football Federation. Participants were excluded if they were currently receiving medical care or had pain during the balance test, but not if they had previous experience with the YBT during rehabilitation or strength and conditioning programs. In addition, six specific positions were analysed: Goalkeeper (GK), defender-fullbacks (DEF), centre back (CEN-B), midfielder (MID), winger (WG) and forward (FW) [1]. All participants were carefully informed of the experimental procedures and possible risks and benefits associated while participating in the study. On the one hand, participants older 18 year of age sign an informed consent document. On the other hand, informed consent for participants who are minors were performed by parents or guardian. Both, before any of the test were performed. The study was conducted in accordance with the ethical principles of the 1964 Helsinki declaration for human research and was approved by the Research Ethics Committee of the Pontifical University of Comillas (2021/74).

### Instruments and procedure

Data from this study were collected at multiple locations using a standardised protocol as part of a pre-season assessment of musculoskeletal function in male soccer players [14]. The measurement of the length of both lower limbs was performed following the test normalization and previous studies [24, 25], that is, from the anterosuperior iliac spine to the most distal point of the tibial malleolus. A tape measure with one side graduated in centimetres (cm), a length of 2 m and a width of 2 cm (Lufkin W606PM) was used in this protocol. The dominant leg was determined by asking participants which leg they used to strike a ball with the greatest possible force and accuracy. The test was conducted barefoot to facilitate foot placement and eliminate variability caused by using footwear. A YBT Kit (Perform Better, West Warwick, Rhode Island) was used, which consists of three connected cylindrical tubular plastic bars marked in half cm increments. Each bar has a moveable indicator plate, which the subject moves by pushing with their foot/toes without bearing weight on the indicator [26]. The player was instructed to stand on the evaluated leg in the center of the platform with the most distal end of the longest toe just behind the red line. While maintaining single leg stand, the player was instructed to reach three trials with the free limb in the anterior direction (AN), posteromedial direction (PM) and posterolateral direction (PL), all designated in relation to the supporting foot, according to the YBT-LQ protocol [26]. The player had to place his hands on the waist to facilitate the observer's control, since if the subject lost balance, it was easily identifiable when the hands were released from the waist. The assessment was performed with both legs (dominant [Dom] and non-dominant [NDom]). All subjects were familiarised with the test, performing it three times with each leg and selecting the maximum value [14, 26, 27].

Attending the study by Falces-Prieto et al. [14], the attempt was considered invalid and repeated if the player: released hands at the hips; moved or lifted the supporting foot at any time during the test; placed the free foot on the ground; lost balance from leaving the starting position until returning back; and was unable to maintain the starting position for at least one second after returning back. If the attempt was unsuccessful, the player moved back to the starting position and the attempt was repeated. If the attempt was successful, when the participant returned to the centre, the result was registered.

### Statistical analysis

Descriptive statistics were performed on all subjects participating in the study, presented as mean ± standard deviation (SD) or percentages. Data distribution was examined for normality using Kolmogorov–Smirnov test (> 50 samples). The root data from the three directions (AN, PM and PL) were normalized by leg length for further analysis, following the formula: Normalized maximal reach distance (% leg length [LL]) = (maximal reach distance [cm])/LL [cm]) × 100. In addition, for the calculation of the composite score, the following formula was used: CS (%) = ((AN + PM + PL [cm])/(LL [cm] × 3)) × 100, where a percentage < 94% is associated with lower limb injury risk. The differences between three possible directions (ANT, PM and PL), in both Dom and NDom leg, as well as the inter-limb asymmetry (Dom leg − NDom leg value) between legs (≥ 4 cm as indicator of injury risk), were analysed employing MANOVA and MANCOVA, taking into consideration five different categories and six field positions, as well as analysing the possible interaction of age (simple variable or multilevel -ranged- variable) as covariable. Finally, multiple pairwise comparisons were employed for obtaining differences within each direction (AN, PM, PL for Dom and NDom leg) by category and/or field position, and the Bonferroni correction was used to compensate the multiple post hoc comparisons. The significance level was set at 5% (*p* < 0.05). The effect size (*d*) was calculated through Cohen's *d* [28]. The interpretation of the d regardless of the sign, followed the scale: very small (0.01), small (0.20), medium (0.50), large (0.80), very large (1.20), huge (2.0) as initially suggested by Cohen [29] and expanded by Sawilowsky [30]. Statistical analyses were performed using SPSS v.26 for Windows (SPSS Inc., Chicago, IL).

## Results

**Fig. 1:**
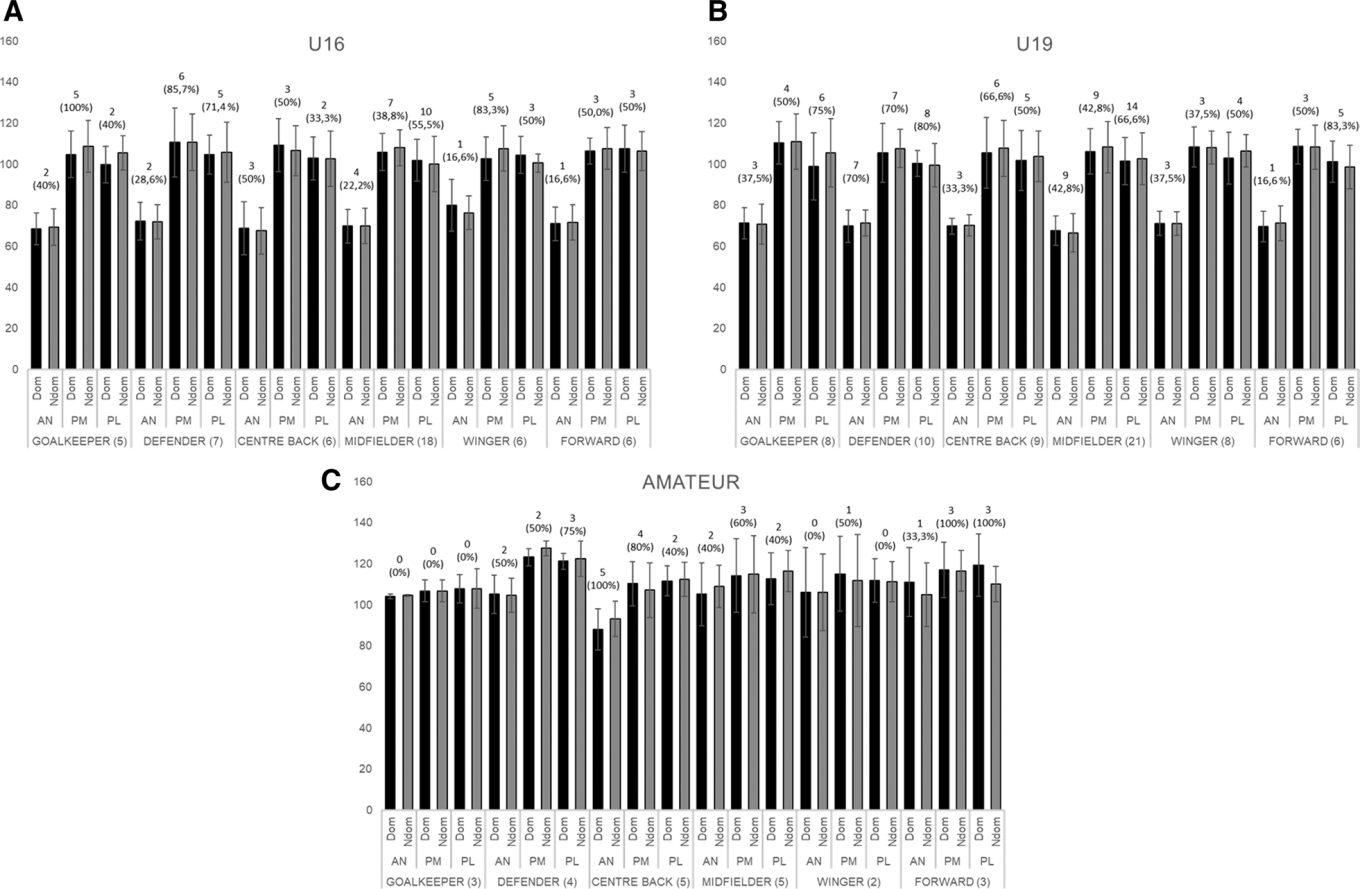
Normalized values for ANT, PM and PL by category (**A** U16, **B** U19 and **C** amateur) and field position, including the number and percentage of players ≥ 4 cm in inter-limb asymmetry

**Fig. 2:**
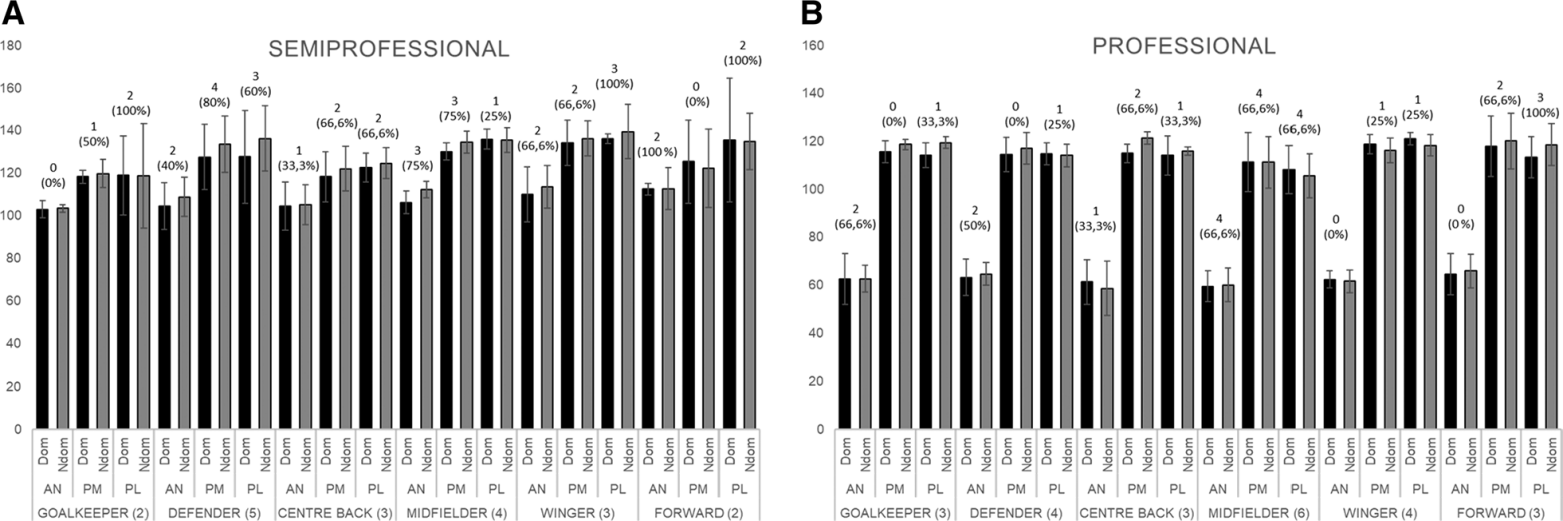
Normalized values for ANT, PM and PL by category (**A** semiprofessional, and **B** professional) and field position, including the number and percentage of players ≥ 4 cm in inter-limb asymmetry

After analysing the possible interaction of age (simple variable or multilevel variable) as covariable, no significant interactions were found and no further analysis were performed. All descriptive results concerning the three directions (normalized values) by category or level and field position are graphically shown in Fig. 1 [Fig. 1A (U16), Fig. 1B (U19) and Fig. 1C (amateur)] and Fig. 2 ([Fig. 2A (Semiprofessional) and Fig. 2B (Professional)]. The number and percentage of players ≥ 4 cm in inter-limb asymmetry is included as indicator of injury risk.

All details concerning the descriptive data of the normalized values (%) for the three directions (AN, PM and PL) in dominant and non-dominant legs by field position and category, the inter-limb asymmetry (cm) and the composite score (AN, PM and PL) (%) are reported in Table 1.

**Table 1:** Normalized values (%) for dominant and non-dominant leg (mean ± SD), Inter-limb asymmetry in cm (ILA) (mean ± SD) and composite score (%) for dominant and non-dominant leg by field position in all categories

Field position	Category	Dom leg			Ndom leg			ILA			CS
		AN	PM	PL	AN	PM	PL	AN	PM	PL	Dom–Ndom
GK											
	U16	68.4 ± 7.7	104.7 ± 11.3	99.7 ± 9.0	69.2 ± 8.9	108. 7 ± 12.6	105.5 ± 8.3	4.4 ± 1.9	11.8 ± 5.9	5.2 ± 5.0	90.5–94.4
	U19	71.2 ± 7.6	110.4 ± 10.3	98.9 ± 16.3	70.6 ± 9.8	110.9 ± 13.5	105.3 ± 16.7	3.6 ± 4.0	4.1 ± 3.4	12.3 ± 11.6	93.5–95.6
	Amateur	104.0 ± 1.2	106.7 ± 5.3	107.7 ± 6.8	104.5 ± 0.4	106.7 ± 5.4	107.9 ± 9.7	0.0 ± 0.0	0.5 ± 0.7	1.5 ± 0.7	106.1–106.4
	SPRO	102.9 ± 4.1	118.2 ± 3.1	118.9 ± 18.6	103.3 ± 1.9	119.6 ± 6.6	118.5 ± 24.6	1.5 ± 0.7	2.5 ± 2.1	4.0 ± 0.0	113.3–113.8
	PRO	62.3 ± 10.6	115.4 ± 4.6	114.0 ± 5.1	62.5 ± 5.6	118.6 ± 2.2	119.3 ± 2.5	3.7 ± 3.5	1.0 ± 1.7	3.7 ± 3.8	97.2–100.1
DEF											
	U16	72.0 ± 9.2	110.5 ± 16.8	104.5 ± 9.43	71.9 ± 8.2	110.6 ± 13.8	105.8 ± 14.6	3.7 ± 2.8	6.3 ± 2.6	6.4 ± 4.5	95.7–96.1
	U19	69.7 ± 7.9	105.4 ± 14.4	100.3 ± 6.2	71.2 ± 6.4	107.6 ± 9.3	99.4 ± 10.6	5.1 ± 2.9	8.7 ± 8.5	7.3 ± 4.3	91.8–92.7
	Amateur	105.0 ± 9.3	123.2 ± 4.1	121.1 ± 3.8	104.7 ± 8.4	127.4 ± 3.6	122.3 ± 8.7	2.7 ± 2.2	4.7 ± 4.8	4.2 ± 2.4	116.4–118.1
	SPRO	104.4 ± 10.9	127.4 ± 15.3	127.6 ± 21.8	108.7 ± 9.2	133.4 ± 13.3	136.2 ± 15.2	3.4 ± 2.1	6.2 ± 3.6	7.8 ± 9.5	119.8–126.1
	PRO	63.1 ± 7.6	114.3 ± 7.2	114.6 ± 4.6	64.4 ± 4.7	117.0 ± 6.6	114.0 ± 4.7	4.0 ± 3.6	1.0 ± 1.1	2.5 ± 1.3	97.3–98.5
CEN-B											
	U16	68.7 ± 13.0	109.1 ± 13.0	102.7 ± 10.4	67.5 ± 11.3	106.5 ± 12.1	102.5 ± 13.4	2.7 ± 2.9	3.7 ± 3.5	3.3 ± 2.6	93.5–92.1
	U19	69.3 ± 3.8	103.6 ± 17.0	100.6 ± 15.2	70.4 ± 5.4	106.5 ± 14.1	103.1 ± 12.9	2.4 ± 1.6	3.9 ± 2.8	6.9 ± 6.9	91.2–93.4
	Amateur	88.0 ± 10.1	110.2 ± 10.8	111.5 ± 7.3	93.0 ± 8.6	107.0 ± 13.3	112.3 ± 8.3	9.0 ± 6.2	5.8 ± 2.9	3.0 ± 2.5	103.2–104.1
	SPRO	104.4 ± 11.4	118.1 ± 11.7	122.4 ± 6.7	105.1 ± 9.4	122.0 ± 10.4	124.5 ± 7.3	2.7 ± 1.5	3.3 ± 2.9	5.0 ± 5.0	115.0–117.2
	PRO	61.1 ± 9.3	114.8 ± 3.9	113.9 ± 8.1	58.4 ± 11.3	121.2 ± 2.6	115.7 ± 1.8	4.0 ± 3.6	7.0 ± 4.6	3.3 ± 4.9	96.6–98.4
MID											
	U16	69.8 ± 8.2	105.8 ± 9.0	101.8 ± 10.2	69.7 ± 8.6	108.0 ± 8.7	100.0 ± 13.5	2.6 ± 2.1	2.9 ± 2.8	6.1 ± 6.3	92.5–92.6
	U19	67.8 ± 7.1	106.9 ± 11.3	101.9 ± 11.4	66.6 ± 9.0	108.6 ± 12.3	102.9 ± 12.3	3.7 ± 2.8	4.8 ± 4.4	5.8 ± 4.6	92.2–92.7
	Amateur	105.0 ± 15.3	114.1 ± 17.9	112.7 ± 12.6	108.8 ± 10.3	114.8 ± 18.7	116.3 ± 10.1	5.8 ± 6.3	10.2 ± 9.7	4.0 ± 4.1	110.6–113.3
	SPRO	106.1 ± 5.2	130.1 ± 4.1	135.8 ± 4.6	112.1 ± 4.0	134.6 ± 5.2	135.4 ± 6.0	5.2 ± 2.2	4.0 ± 2.0	3.2 ± 2.6	124.0–127.4
	PRO	59.2 ± 6.4	111.1 ± 12.4	107.9 ± 10.1	59.9 ± 6.9	111.1 ± 10.8	105.3 ± 9.2	4.2 ± 2.2	4.8 ± 3.1	5.5 ± 4.0	92.8–92.1
WG											
	U16	79.8 ± 12.5	102.5 ± 10.7	104.4 ± 9.1	76.2 ± 8.2	107.5 ± 11.1	100.4 ± 4.4	4.0 ± 6.5	8.5 ± 4.1	5.3 ± 5.2	95.6–94.7
	U19	71.1 ± 6.0	108.3 ± 9.8	102.9 ± 12.6	71.1 ± 5.7	107.9 ± 8.1	106.4 ± 7.9	3.2 ± 2.5	4.2 ± 4.6	6.0 ± 6.3	94.1–95.1
	Amateur	106.1 ± 21.8	115.0 ± 18.3	111.8 ± 10.7	105.9 ± 18.7	111.7 ± 22.5	111.1 ± 9.8	1.5 ± 0.7	3.5 ± 3.5	0.0 ± 0.0	94.1–95.1
	SPRO	109.8 ± 13.0	134.1 ± 10.6	136.2 ± 2.2	113.5 ± 9.9	136.1 ± 8.3	139.4 ± 12.7	3.0 ± 2.6	5.0 ± 5.0	7.3 ± 2.5	126.7–129.7
	PRO	62.1 ± 3.6	118.6 ± 4.1	120.9 ± 2.5	61.4 ± 4.7	116.0 ± 5.2	118.2 ± 4.4	2.0 ± 0.8	1.7 ± 1.7	1.7 ± 1.7	100.5–98.5
FW											
	U16	70.9 ± 8.1	106.2 ± 6.3	107.5 ± 11.6	71.5 ± 8.6	107.6 ± 10.3	106.3 ± 9.5	2.7 ± 1.9	4.5 ± 4.1	6.0 ± 4.9	94.8–93.5
	U19	69.6 ± 7.4	108.5 ± 8.6	101.2 ± 10.0	71.2 ± 8.6	108.3 ± 10.8	98.5 ± 10.6	1.8 ± 2.6	4.5 ± 3.7	6.3 ± 4.7	93.1–92.6
	Amateur	110.9 ± 16.8	117.0 ± 13.5	119.2 ± 15.1	104.8 ± 15.6	116.5 ± 9.9	110.0 ± 8.5	5.3 ± 8.4	6.0 ± 2.0	12.0 ± 9.5	115.7–110.4
	SPRO	112.3 ± 2.8	125.2 ± 19.4	135.4 ± 28.9	112.6 ± 9.7	122.1 ± 18.3	134.7 ± 13.3	4.0 ± 0.0	2.5 ± 0.7	9.0 ± 0.0	124.3–123.1
	PRO	64.3 ± 8.5	117.7 ± 12.6	113.1 ± 8.5	65.7 ± 7.1	119.9 ± 11.6	118.4 ± 8.7	1.7 ± 1.1	3.7 ± 0.6	4.3 ± 0.6	98.4–101.4

Data is presented as mean ± SD. The units are: % for AN, PM and PL normalized values; % for composite score (CS); cm for Inter-limb asymmetry (ILA)

### YBT by category or level

Taking into consideration the overall data, significant differences were found for AN, PM and PL in both dominant and non-dominant legs. For AN movement (Dom and NDom legs), the highest values were reported by amateurs and SPRO (Range = 103.24–109.21%), and the lowest level in PRO players (62.0%). Significant differences were found in Dom and NDom legs, where U16 reported a lower distance covered compared to amateur (− 31.62% Dom [*d* =  − 2.52] and − 32.60% NDom [*d* =  − 3.22]) and SPRO (− 35.11% Dom [*d* =  − 3.81] and − 38.22% NDom [*d* =  − 4.67]) but higher compared to PRO (+ 9.64% Dom [*d* = 1.13] and + 8.95% NDom [*d* = 1.15]) (*p* < 0.001 for all comparisons). Similar results were reported in U19 players (*p* < 0.001). Amateur (+ 41.14% Dom [*d* = 3.61] and + 41.56% NDom [*d* = 4.52]; *p* < 0.001) and SPRO (+ 44.63% Dom [*d* = 5.72] and + 47.15% NDom [*d* = 6.65]; *p* < 0.001) categories showed significant higher values compared to PRO category as well.

Regarding PM movement for both legs, highest values were reported by SPRO category (125.52–127.97% Dom-NDom, respectively) followed by PRO (115.34–117.28%, Dom-NDom) and amateurs (114.41–114.03%, Dom-NDom). Significant differences were found between U16 and amateur (− 7.90% Dom [*d* =  − 0.71]; *p* < 0.05) SPRO (− 19.16% Dom [*d* =  − 1.77]; − 19.88% NDom [*d* =  − 1.98]; *p* < 0.001) and PRO categories (− 8.93% Dom [*d* =  − 0.87]; − 9.11 NDom [*d* =  − 0.89]; *p* < 0.01). Similar results were found in U19 players (*p* < 0.01 or *p* < 0.001, depending on comparisons). Likewise, differences between Amateur and SPRO (− 11.20% Dom [*d* =  − 0.97] and − 13.97% NDom [*d* =  − 1.18]; *p* < 0.01 or *p* < 0.001, respectively) and SPRO and PRO (+ 10.20% Dom [*d* = 1.13] and + 10.72% NDom [*d* = 1.35]; *p* < 0.01) were found.

The PL values showed significant differences between all categories for Dom and NDom leg ([*d*_range_ =  − 1.08 to − 2.42], *p* < 0.01–*p* < 0.001, depending on the multiple comparisons), except for the pairwise U16-U19 and amateur-PRO. The highest value was registered for SPRO category for both legs (129.42–131.53% for Dom and NDom leg, respectively), followed by PRO and amateur, and finally the lowest age categories.

Concerning the inter-limb asymmetry, the highest value was observed in amateurs (AN = 0.04 m), U16 (PM = 0.06 m) and U19 (PL = 0.07 m). Significant differences were found only for PM (pairwise U16-PRO [*d* = 0.56]; *p* < 0.01) and PL (pairwise U19-amateur [*d* = 0.54], *p* < 0.05; U19-PRO [*d* = 0.63], *p* < 0.01).

### YBT by field position

On the other hand, analysing the overall data by field position, only significant differences were found for AN Dom and NDom leg between CEN-B position – WG (− 7.51% Dom [*d* =  − 0.23], *p* < 0.01; − 6.72% NDom [*d* =  − 0.14]; *p* < 0.05) CEN-B – FW (− 7.33% Dom [*d* =  − 0.20]; − 6.34% NDom [*d* =  − 0.16]; *p* < 0.05), and DEF – CEN-B (+ 5.36% NDom [*d* = 0.24]; *p* < 0.05). No significant differences por PM, PL and inter-limb asymmetry was found regarding field position.

### Differences between age categories within each field position, YBT direction (ANT, PM, PL) and inter-limb asymmetry

#### AN direction

Concerning specific field positions, within the GK position, significant differences were found between U16-amateur (Dom [*d* =  − 6.44] and NDom [*d* =  − 5.62], *p* < 0.001); U16-SPRO (Dom [*d* =  − 5.59] and NDom [*d* =  − 5.30], *p* < 0.001); U19-amateur (Dom [*d* =  − 6.05] and NDom [*d* =  − 4.86], *p* < 0.001); U19-SPRO (Dom [*d* =  − 5.21] and NDom [*d* =  − 4.60], *p* < 0.001); Amateur-PRO (Dom [*d* = 5.52] and NDom [*d* = 10.56], *p* < 0.001); SPRO-PRO (Dom [*d* = 5.05] and NDom [*d* = 9.74], *p* < 0.001). Very similar differences between those pairwise in Dom and NDom legs were found in the rest of field positions.

#### PM direction

The field positions with a higher number of significant differences between categories were DEF, MID and WG. For DEF, significant differences were found between lower categories U16 and U19 respect to amateurs (NDom leg, [*d* =  − 1.67 and − 2.82, respectively], *p* < 0.05 or *p* < 0.01, respectively), as well as SPRO category (for both legs, [*d*_range_ =  − 1.05 to − 2.26], *p* < 0.01 or *p* < 0.001, for U16 and U19, respectively). Likewise, differences between SPRO-PRO (NDom leg, [*d* = 1.57], *p* < 0.05) were reported. In the same way, for MID, differences were established between lower categories U16-U19 and SPRO (both legs, [*d*_range_ =  − 2.71 to − 3.71], *p* < 0.001); amateur and SPRO (NDom, [*d* =  − 1.43], *p* < 0.05); or PRO (Dom, [*d* = 0.19], *p* < 0.05); as well as between SPRO and PRO (both legs, [*d* = 2.04–2.77 for Dom and NDom, respectively], *p* < 0.01). The WG showed closer differences between categories that those reported by the previous described field positions.

#### PL direction

Multiple significant differences between categories were found for several field positions. Differences between the categories U16-SPRO and U19-SPRO are common to the six field positions (both legs for all positions, except for GK being only Dom leg [*d*_range_ =  − 0.63 to − 4.81]; *p* < 0.05, *p* < 0.01 or *p* < 0.001, depending on the field positions). In addition, significant differences were shown for lower categories U16 and U19 respect to amateur (DEF and MID [*d*_range_ =  − 1.19 to − 4.02]; *p* < 0.05, or *p* < 0.01, depending of the cases) and PRO categories (DEF, WG and FW [*d*_range_ =  − 1.78 to − 4.01]; *p* < 0.05). Finally, in four field positions (DEF, MID, WG and FW) significant differences were reported for the pairwise amateur-SPRO (both legs except for FW position -NDom- [*d*_range_ =  − 2.21 to − 3.15]; *p* < 0.05 or *p* < 0.01) and SPRO-PRO (NDom leg except MID with both legs [*d*_range_ = 1.45–3.87]; *p* < 0.05 or *p* < 0.01).

#### Inter-limb asymmetry (AN, PM, PL)

Only significant differences were found in AN within CEN-B position, specifically between U16 and U19 respect to amateur ([*d* =  − 1.26 and − 1.56]; *p* < 0.05 and *p* < 0.01, respectively) and between amateur and SPRO ([*d* = 1.34]; *p* < 0.01) or PRO ([*d* = 0.98]; *p* < 0.05). A higher number of differences were found in PM, highlighting the GK showing differences between U16 and the rest of categories ([*d*_range_ = 1.69–2.56]; *p* < 0.01), MID with differences between U16-19 categories and amateur ([*d* =  − 0.66 and − 0.95]; *p* < 0.05), as well as amateur and SPRO ([*d* = 0.83], *p* < 0.05) or PRO ([*d* = 0.68]; *p* < 0.05) and FW with differences between U16-PRO ([*d* = 0.34]; *p* < 0.05). Only the GK position showed differences in PL, between U16-19 [*d* =  − 0.76], U19-amateur [*d* = 1.17] or U19-PRO [*d* = 0.89] (*p* < 0.05 for all cases).

### Differences between field positions within each age category, YBT direction (ANT, PM, PL) and inter-limb asymmetry

#### AN direction

Only differences for U16 (WG vs. GK, CEN-B and MID for Dom leg [*d*_range_ =  − 0.87 to − 1.10]; *p* < 0.05) and Amateur (CEN-B vs. GK, WG and FW for Dom leg [*d*_range_ =  − 1.06–2.23], *p* < 0.05; CEN-B vs. DEF and MID for Dom [*d* = 1.76 and − 1.31, respectively], *p* < 0.01 and NDom [*d* = 1.37 and − 1.66, respectively], *p* < 0.05) were reported.

#### PM and PL directions

Differences for amateur category were found in PM for DEF versus GK and CEN-B (NDom [*d* =  − 4.53 and 2.09, respectively]; *p* < 0.05). For PL, only SPRO category showed differences (GK-WG, NDom [*d* =  − 1.07]; *p* < 0.05).

#### Inter-limb asymmetry (AN, PM, PL)

Differences in AN for amateur category were found between CEN-B versus GK, DEF and WG ([*d*_range_ =  − 1.34 to − 2.12]; *p* < 0.01), and GK-MID ([*d* =  − 1.41]; *p* < 0.01). Concerning PM, three categories showed differences in field positions. U16 (GK vs. the rest of positions, [*d*_range_ = 1.26–1.90]; *p* < 0.01); U19 (DEF vs. GK, CEN-B, MID and WG ([*d* = 0.57–0.74]; *p* < 0.05); amateur (GK-MID ([*d* =  − 1.27]; *p* < 0.01). Finally, for PL inter-limb asymmetry only U19 category (GK vs. CEN-B, MID, WG and FW, [*d*_range_ = 0.51–0.65]; *p* < 0.05) and amateur (FW vs. GK, CEN-B, MID and WG, [*d*_range_ =  − 1.05 to − 1.70]; *p* < 0.05) reported significant differences.

#### Bivariate correlations

Correlations between the AN, PM, PL directions, inter-limb asymmetry and other variables such as category, height and age groups are shown in Table 2.

**Table 2:** Bivariate correlations for ANT, PM, PL directions, Inter-limb asymmetry as well as categorical variables (category or level. height and age groups)

N = 173		YBT-AN Dom leg	YBT-PM Dom leg	YBT-PL Dom leg	YBT-AN NDom leg	YBT-PM NDom leg	YBT-PL NDom leg	Inter-limb asymmetry AN	Inter-limb asymmetry PM	Inter-limb asymmetry PL	Category or level	Height	Age groups
YBT-AN Dom leg	Pearson correlation	1	.520**	.571**	.954**	.525**	.597**	.042	.070	− .009	.212**	.223**	.245**
	Sig. (bilateral)		.000	.000	.000	.000	.000	.583	.359	.902	.005	.003	.001
YBT-PM Dom leg	Pearson correlation	.520**	1	.777**	.484**	.818**	.733**	− .021	− .202**	− .169*	.386**	.196**	.333**
	Sig. (bilateral)	.000		.000	.000	.000	.000	.781	.008	.026	.000	.010	.000
YBT-PL Dom leg	Pearson correlation	.571**	.777**	1	.578**	.774**	.804**	.026	− .028	− .209**	.476**	.198**	.424**
	Sig. (bilateral)	.000	.000		.000	.000	.000	.732	.715	.006	.000	.009	.000
YBT-AN NDom leg	Pearson correlation	.954**	.484**	.578**	1	.549**	.633**	.075	.105	− .040	.240**	.209**	.266**
	Sig. (bilateral)	.000	.000	.000		.000	.000	.324	.171	.601	.002	.006	.000
YBT-PM NDom leg	Pearson correlation	.525**	.818**	.774**	.549**	1	.802**	.044	.010	− .168*	.394**	.192*	.328**
	Sig. (bilateral)	.000	.000	.000	.000		.000	.568	.894	.027	.000	.011	.000
YBT-PL NDom leg	Pearson correlation	.597**	.733**	.804**	.633**	.802**	1	.041	.008	− .153*	.486**	.228**	.430**
	Sig. (bilateral)	.000	.000	.000	.000	.000		.594	.922	.044	.000	.003	.000
Inter-limb asymmetry AN	Pearson correlation	.042	− .021	.026	.075	.044	.041	1	.311**	.103	.042	.063	.063
	Sig. (bilateral)	.583	.781	.732	.324	.568	.594		.000	.179	.582	.411	.408
Inter-limb asymmetry PM	Pearson correlation	.070	− .202**	− .028	.105	.010	.008	.311**	1	.072	− .127	− .044	− .078
	Sig. (bilateral)	.359	.008	.715	.171	.894	.922	.000		.345	.097	.562	.305
Inter-limb asymmetry PL	Pearson correlation	− .009	− .169*	− .209**	− .040	− .168*	− .153*	.103	.072	1	− .121	− .075	− .125
	Sig. (bilateral)	.902	.026	.006	.601	.027	.044	.179	.345		.113	.328	.101
Category or level	Pearson correlation	.212**	.386**	.476**	.240**	.394**	.486**	.042	− .127	− .121	1	.390**	.949**
	Sig. (bilateral)	.005	.000	.000	.002	.000	.000	.582	.097	.113		.000	.000
Height	Pearson correlation	.223**	.196**	.198**	.209**	.192*	.228**	.063	− .044	− .075	.390**	1	.399**
	Sig. (bilateral)	.003	.010	.009	.006	.011	.003	.411	.562	.328	.000		.000
Age groups	Pearson correlation	.245**	.333**	.424**	.266**	.328**	.430**	.063	− .078	− .125	.949**	.399**	1
	Sig. (bilateral)	.001	.000	.000	.000	.000	.000	.408	.305	.101	.000	.000	

**The correlation is significant at the 0.01 level (bilateral)

*The correlation is significant at the 0.05 level (bilateral)

## Discussion

The purpose of the current cross-sectional study was to analyze the variations of dynamic balance between under-16, under-19, amateurs, semi-professional and professional men soccer players, also considering playing positions. The main results of this study revealed that for both, dominant and non-dominant legs, older players tended to have better performance with exception of anterior dynamic balance in which professionals were the worst. Regarding inter-limb asymmetry, no meaningful differences were found between players.

Although clear limitations in the evidence found about dynamic balance in Y-balance test between competitive levels in different team sports [2–4], the results found in the present study are contradictory regarding the unique conducted so far in soccer [5]. In the cross-sectional study conducted in 38 high-school, 37 college, and 44 professional men soccer players, the results suggested a significant tendency for better results of professionals in posteromedial and posterolateral reach directions, while high-school athletes performed significantly better in anterior reach [31]. In our study, professionals were meaningfully worst in anterior reach in comparison to younger and semi-professional and amateurs. Additionally, in our study older players were better in posterolateral and posteromedial, although professionals were worse than semi-professional and adult amateurs.

Despite dynamic balance be dependent from different factors (e.g., range of motion, sex, movement abilities, strength and proprioception) [3, 10, 32–34], it would be expectable to observe some sensitivity of the test to different competitive levels, but hypothesizing a favorable tendency for professional players. However, in the current study, it was found that semi-professional players had significant better scores in both dominant and non-dominant legs. This may be caused by different factors, not properly researched in the current article. Despite some evidence suggesting better physical fitness levels of professionals, namely regarding strength [35] and differences in match load demands considering the competitive levels [36], our findings was in the opposite way. One of the explanations can be related with similar frequency of training sessions and matches of both groups. Additionally, non-observed factors as strength or range of motion levels were not considered and those may be justification for the findings in the present article. Finally, the report of previous injuries was not considered, which may influence the dynamic balance considering previous research [37].

On the other side, and in line with previous study [31], youngers were worse than adults in posteromedial and posterolateral reach, maybe justified by the lower stability of youngers or even for the lowest hip abduction strength levels, since is one of the factors that is significantly associated with higher scores in YBT [10].

Comparisons between playing positions revealed that center backs were worse than wingers and forwards. Although limited reports about that, a study conducted in elite soccer players revealed that strikers had significant greater composite scores in YBT in comparison to midfielders or defenders [14]. No in line with that, a study conducted in professional Turkish players revealed no significant differences between playing positions in the different Y-balance scores [11]. Differences could be expectable considering the type of movements made by players. Eventually, since wingers and forwards may perform more sprints, this may lead to higher mobility patterns that may eventually contribute to better performance scores in comparison to centre-backs that perform more accelerations, which have less range of movement [38, 39].

In the current research was also found that anterior scores were the worse obtained comparing to posteromedial and posterolateral values. This finding was confirmed for both semi-professional and professionals. Such fact is in line with a previous study in soccer that reported 4% less score in anterior balance [5]. Regarding the inter-limb asymmetry there was no evidence of differences among competitive levels [40], thus suggesting that despite both were different in Y-balance scores, such fact did not compromise the asymmetry between limbs in each level.

One of the limitations of the current study was the non-report of determinant co-variables (e.g., strength, range of motion) of players that may helped to explain the evidence of differences between professionals and semi-professional players. Additionally, the use of only one team for each competitive level must be carefully considered in the interpretation of the results, since are not representative of competitive levels. Future studies should increase the number of teams involved and sample size for increasing the possibility of generalization of the findings. Additionally, inclusion of covariables as strength and range of motion levels should be considered to explain the variations.

As practical implications, we should emphasize that balance-based interventions could be implemented in a more structured way, namely using different approaches such as strength training focusing on stability and balance, the use of unilateral exercises for increasing the ability of balance without asymmetries and reinforcing the assessment of balance ability in teams aiming to individualize the training process.

## Conclusions

In brief, the findings of this study should be carefully considered for eventual generalization. As reported in a comparative study in volleyball, the differences in scores can be justified not by the competitive level but the training programs implemented in the teams. Therefore, the main practical implications extractable from this study is that eventual considerations about training programmes and co-variables should be considered for explaining variations in dynamic balance between competitive levels within the same age-group (Additional files 1 and 2).

## Supplementary Information

**Additional file 1:** Supplementary file 1.

**Additional file 2:** Supplementary file 2.

## Data Availability

The datasets generated during and analyzed during the current study are available from the corresponding author on reasonable request.

## Abbreviations

YBT: Y-Balance-Test
ANT: Anterior
PM: Posteromedial
PL: Posterolateral
Dom: Dominant
NDom: Non-dominant
SEBT: Star Excursion Balance Test
SPRO: Semi-professional
PRO: Professional
GK: Goalkeeper
DEF: Defender (fullbacks)
CEN-B: Centre back
MID: Midfielder
WG: Winger
FW: Forward
